# Effect of Processing and *In Vitro* Digestion on Bioactive Constituents of Powdered IV Range Carrot (*Daucus carota*, L.) Wastes

**DOI:** 10.3390/foods12040731

**Published:** 2023-02-07

**Authors:** Claudia Bas-Bellver, Cristina Barrera, Noelia Betoret, Lucía Seguí

**Affiliations:** Instituto Universitario de Ingeniería de Alimentos para el Desarrollo, Universitat Politècnica de València, 46022 Valencia, Spain

**Keywords:** carrot, waste recovery, carotenoids, antioxidant properties, functional powders, *in vitro* digestion

## Abstract

*Daucus carota* L. is an important food crop utilized worldwide and a rich source of bioactive compounds. Carrot processing generates residues which are discarded or underused, for which using them as a source for obtaining new ingredients or products is an opportunity for the development of healthier and more sustainable diets. In the present study, the impact of different milling and drying procedures and *in vitro* digestion on the functional properties of carrot waste powders was evaluated. Carrot waste was transformed into powders by disruption (grinding vs. chopping), drying (freeze-drying or air-drying at 60 or 70 °C) and final milling. Powders were characterized in terms of physicochemical properties (water activity, moisture content, total soluble solids and particle size) nutraceuticals (total phenol content, total flavonoid content antioxidant activity by DPPH and ABTS methods, as well as carotenoid content (α-carotene, β-carotene, lutein, lycopene). Antioxidants and carotenoid content during *in vitro* gastrointestinal digestion were also evaluated; the latter in different matrices (directly, in water, in oil, and in oil-in-water emulsion). Processing allowed to reduce water activity of samples and obtain powders rich in antioxidant compounds and carotenoids. Both disruption and drying had a significant impact on powders’ properties freeze-drying led to finer powders with higher carotenoid content but lower antioxidant values, whereas air-drying implied chopped air-dried powders exhibited higher phenols content and improved antioxidant activity. Simulated *in vitro* digestion studies revealed that digestion helps release bioactive compounds which are bound to the powder structure. The solubilization of carotenoids in oil was low, but fat co-ingestion notably increased their recovery. According to the results, carrot waste powders containing bioactive compounds could be proposed as functional ingredients to increase the nutritional value of foods, thus contributing to the concepts of more sustainable food systems and sustainable healthy diets.

## 1. Introduction

Carrot (*Daucus carota* L.) is a root crop of the *Umbelliferae* family. From a nutritional point of view, it is a rich source of bioactive compounds such as vitamins, minerals, antioxidant compounds and dietary fibre [1,2]. Orange carrots are a well-known source of phenolics and α- and β-carotene, which impart its characteristic colour and account for about half of the provitamin A carotenoid found in the food supply [1,2,3,4]. *Daucus carota* is an important food crop utilized worldwide which can be consumed raw, in juice or drinks, cooked as a savoury dish or in sweet dishes [2]. However, as it is the case of most vegetables, carrot processing generates by-products, such as culled carrots and carrot waste.

A variety of technologies are aimed at adding value to vegetables and fruit residues and reduce their environmental impact [5]. These include chemical, physical, and biological transformations applied alone or combined to produce functional components, novel foods, extracted chemicals, biofuels or pomace powder, among others [2]. Carrot residues can be successfully stored in their dried form, which can be used in the development of bakery products and extrudates [2], and also as an ingredient in prepared foods such as instant soups or healthy snack foods [6]. When properly managed, dehydration can be applied to obtain powders from vegetable wastes, greatly preserving the bioactive compounds of interest, thus broadening the benefits these by-products can offer as a source of antioxidants, antimicrobial compounds and other bioactive substances [7,8]. This makes it really interesting to obtain powdered products based on carrots to use them as functional ingredients in food formulation. Among dehydration techniques, hot-air drying (HAD) and freeze-drying (FD) are usually applied in the food industry to reduce water activity to levels that guarantee product stability and extend shelf life [9], in addition to obtaining a final product that is easier to store, transport, dose and mix with other foods [1]. HAD is simpler and more economic, but implies exposure to conditions which promote oxidation, crusting and loss of organoleptic and functional properties [10]; in contrast, FD prevents oxidation during processing, minimizes compositional changes and largely preserves volatile and soluble compounds, yielding a final porous structure with a minimum moisture content, but at a higher cost and energy requirements [10].

Carrots contain a variety of bioactive compounds, including flavonoids, phenolic acids, carotenoids, polyacetylenes, and ascorbic acid. Among the bioactive constituents of carrots, carotenoids are probably the most important and the most studied ones, although polyacetylenes have also raised significant interest in recent years due to their anti-tumour and anti-inflammatory properties [11]. Carotenoids are lipophilic pigments synthesized mainly in plants, and also microorganisms, but not by animals [12,13]. They are a group of yellow, orange, and red phytochemicals [3], which are classified as nonpolar carotenes (composed exclusively of carbon and hydrogen) and less nonpolar xanthophylls (with at least one functional group containing oxygen), based on structural differences [12]. Carotenoids are very unstable compounds, especially once released from the food matrix, since they are highly sensitive to light, oxidation, acid pH and temperature [14]. Hence, harvesting, storage and processing conditions are decisive to preserve them [4].

In addition to the provitamin A activity attributed to some carotenes (β-carotene, α-carotene, β-cryptoxanthin), carotenoids have an important physiological activity contributing to good human health [15]. Thus, they exhibit antioxidant activity, enhance the immune system, protect against solar radiation or help maintain the visual system [13,15,16]. Moreover, carotenoids may reduce the risk of cardiovascular disease, certain types of cancer, and age-related macular degeneration and cataracts [17]. Since humans are not capable of synthesizing them, we can only obtain them through our diets [15,18]. Carotenoids are mainly obtained from fruits and vegetables and are also added to food as colorants. The most widely distributed ones are β-carotene (e.g., carrots, spinach, banana, mandarins) and lutein (mostly obtained from green vegetables) [19,20].

Despite plant foods containing high levels of carotenoids, their bioavailability is rather low, particularly when foods are consumed raw and unprocessed. To exert their health-promoting properties, carotenoids must be released from the food matrix after ingestion and remain stable during the process until being absorbed and finally distributed throughout the body [12,18]. Therefore, in order to improve their health-beneficial effects, vegetables and fruits are better consumed after being cooked or processed, e.g., mechanically and thermally treated, and also co-consumed with lipids [20]. Regarding the latter, the addition of fat plays an important role in carotenoid bioavailability since, besides stimulating the release of bile salts and the enzymes responsible for the micellarization of fat-soluble compounds, it increases the size and number of micelles in the medium, favouring carotenoids solubilization and their absorption by the intestinal cells [21,22]. The type and amount of fat added duringthe digestion process determine the results.

*In vitro* methods simulating digestion processes are widely used to study the gastro-intestinal behaviour of food and pharmaceuticals [23]. Simulated digestion methods generally include the oral, gastric and small intestinal phases, and occasionally large intestinal fermentation. Digestion models are used to assess digestibility and bioaccessibility of macro and micronutrients: i.e., the amount of compound that is released from the matrix and is considered to be available for absorption. *In vitro* simulated digestion methods are used to study the matrix release of micronutrients such as phenolics or carotenoids [24].

The aim of the present work was to evaluate the effect of the process variables on the bioactive constituents’ content of carrot waste powders and their response to *in vitro* gastrointestinal digestion. Impact of previous disruption (grinding or chopping) and the different dehydration methods and conditions applied (hot-air drying at 60 or 70 °C or freeze-drying) on physicochemical properties, phenol and flavonoid contents, antiradical capacity and carotenoids content, were evaluated before and after *in vitro* digestion. Moreover, the effect of dispersing powders in different matrices (water, oil, or oil-in-water emulsion) on the release of individual carotenoids from carrot powders during *in vitro* digestion was also assessed.

## 2. Materials and Methods

### 2.1. Raw Material

Discards from the ready-to-eat carrot sticks processing line were provided by the agricultural cooperative Agrícola Villena, Coop. V. (Alicante, Spain At the cooperative’s facilities), whole carrots (*Daucus carota*, L.) were washed, mechanically peeled and cut into sticks. Sticks not meeting the quality standards in terms of size and shape, as well as small pieces, were discarded, which constituted the raw material for the present study. Discards were shipped to the IIAD (UPV) facilities and stored at 4 °C until their use in the experiments described next. This IV-range carrot-processing line produces around 3000 tons per year of discarded product.

### 2.2. Powders Manufacturing

Carrot discards were disrupted to obtain different particle sizes in a food processor (Thermomix^®^ TM6, Vorwerk, Madrid, Spain). Rotating speed and time were adjusted to obtain chopped (C) or medium size pieces of ≤10 mm diameter (350 g, 5 s at 5000 rpm) and ground (G) or small size pieces of ≤5 mm diameter (350 g, 10 s at 10,000 rpm). Chopped and ground carrot discards were then dehydrated by hot-air drying (HAD) or freeze-drying (FD). HAD was carried out in a convective tray dryer with transversal flux (Pol-eko Aparatura, Katowice, Poland) at 60 or 70 °C until reaching a water activity (a_w_) below 0.3. For that purpose, disrupted wastes were distributed in layers of approximately 1 cm thickness on perforated trays (200 g of sample per tray). Drying at 60 °C implied 16 and 17 h for ground and chopped samples, respectively, whereas drying at 70 °C took 12 h in both cases. As for FD, it was conducted in ground samples, and it required a previous freezing step in a deep freezer (Matek model CVN-40/105) at −40 °C for 24 h). Then, samples were lyophilized in the freeze dryer equipment (LyoAlfa 6-80, Telstar, Terrasa, Spain) for 24 h at 0.1 mbar of pressure and −45 °C (condenser temperature). Once dehydrated, freeze-dried or air-dried samples were milled at 10,000 rpm for 2 min at 30 s intervals (Thermomix^®^ TM6, Vorwerk, Madrid, Spain) in order to obtain a fine-grained powder. The powders obtained were stored in closed and opaque glass jars at room temperature to ensure their conservation until analysis. Conditions for air-drying, freeze-drying and disruption were established according to preliminary tests and previous published research [25].

As a result, 5 different powders were obtained, which will be identified hereinafter by the disruption pre-treatment as G, ground and C, chopped, and by the dehydration technique and conditions applied (HAD60 for hot-air drying at 60 °C, HAD70 for hot-air drying at 70 °C, and FD for freeze-drying).

### 2.3. In Vitro Digestion Method

The in vitro simulation of gastrointestinal digestion (oral, gastric and intestinal phases) was performed according to the standardized INFOGEST method proposed by Minekus et al. [23]. Accordingly, simulated digestive fluids, Simulated Salivary Fluid (SSF), Simulated Gastric Fluid (SGF) and Simulated Intestinal Fluid (SIF), were reproduced. To simulate the oral phase, samples were mixed in a 1:1 ratio (*w*/*v*) with the simulated salivary fluid (SSF) and vortexed (Reax top, Heidolph Instruments GmbH & Co. KG, Schwabach, Germany) for 2 min at 37 °C. To simulate the gastric phase, the oral bolus was mixed in a 1:1 (*v*/*v*) ratio with the simulated gastric fluid (SGF) and kept in constant stirring at 55 rpm (Intell-Mixer RM-2, Elmi Ltd., Riga, Latvia) and at 37 °C (JP Selecta SA, Barcelona, Spain) for 2 h. For the intestinal phase, the chyme was mixed in a 1:1 (*v*/*v*) ratio with the simulated intestinal fluid (SIF) and stirred at 55 rpm (Intell-Mixer RM-2, Elmi Ltd., Riga, Latvia) and 37 °C (JP Selecta SA, Barcelona, Spain) for 2 h more. Sampling for subsequent analysis of total phenols, total flavonoids, total antioxidant activity and carotenoids content was performed at the end of both the gastric and the intestinal phases. Antioxidant properties were determined on the whole digested sample, and on the supernatant and pellets collected after centrifugation at 10,000 rpm for 5 min (Eppendorf^®^ Centrifuge 5804R, Hamburg, Germany). Carotenoid content was exclusively analysed on the whole digested sample, as explained later. When appropriate, the bioaccessibility index (BI) and recovery index (RI) were calculated as follows (Equations (1) and (2)).

(1) $$\mathrm{BI}\left(\%\right)=\frac{A}{B}\cdot 100$$

(2) $$\mathrm{RI}\left(\%\right)=\frac{C}{B}\cdot 100$$

where:

A is the amount (µg) of the compound of interest in the soluble fraction after the intestinal phase of digestion; B is the amount (µg) of the compound of interest in the undigested powder; and C is the amount (µg) of the compound of interest in the total digest after the gastric or the intestinal phase.

BI refers to the percentage of compounds which remain solubilized in the chyme after the intestinal phase with respect to the undigested sample, which approximates the proportion of bioactive compounds available for absorption by the intestinal cells. RI refers to the percentage of compounds present in the total digested sample after the gastric or the intestinal phase of digestion [26,27].

The effect of different dispersing media on the response to simulated in vitro digestion was evaluated by simulating the same digestion procedures with powders dispersed in a 1:1 ratio (*v*/*v*) with the following media: water, sunflower oil, and a 10% (*v*/*v*) oil-in-water emulsion. The latter was obtained by dispersion (Ultra-Turrax^®^ T25D, IKA^®^-Werke GmbH & Co. KG, Staufen, Germany) at 10,000 rpm for 10 min. This part of the experiment was conducted on ground air-dried samples at 70 °C (G_HAD70) and freeze-dried ones (FD), in view of powder characterization results.

### 2.4. Analytical Determinations

#### 2.4.1. Water Activity, Moisture Content, Total Soluble Solids, and Particle Size

Water activity (a_w_) was measured at 25 °C with a dew point hygrometer (Aqualab 4TE, Decagon devices, Inc., Pullman, Washington, WA, USA). Moisture content (xw) was calculated from the weight loss undergone by a specific amount of sample during its drying in a vacuum oven (Vaciotem, JP Selecta SA, Barcelona, Spain) at 10 mm Hg and 60 °C until constant weight [28]. The total soluble solids content (xss) was estimated from the Brix degrees measurement obtained at 20 °C with a thermostated Abbe refractometer NAR-3T (Atago, Tokyo, Japan). When needed, water was added in a 1:10 (*w*/*v*) ratio. Particle size distribution of the powders was obtained by laser diffraction using a Mastersizer 2000 equipment (Malvern Panalytical Ld., Malvern, UK). For the dry method determination, a dispersion unit Sirocco 2000 with air as dispersant at 2.5 bar of pressure and 60% speed was used. For the wet method, the particle absorption index was set at 0.1 and refractive indexes of 1.52 and 1.33 were applied to the sample and the dispersed phase (deionized water), respectively. From the particle size distribution curves, the volume moment mean diameter D [4,3] and the surface area moment mean diameter D [3,2], together with the distribution percentiles d_10_, d_50_ and d_90_, were obtained.

#### 2.4.2. Antioxidant Properties of Carrot Wastes and Powders

Total phenol content, total flavonoid content, and antioxidant activity using the DPPH and ABTS methods were determined. Determinations were carried out on sample extracts obtained from 1 g of fresh carrot waste or 0.5 g of non-digested powder or digested precipitate, with 10 mL of an 80% (*v*/*v*) methanol/water solution. Extraction was performed using a horizontal stirrer (Magna Equipments S. L., model ANC10, Barcelona, Spain) in dark conditions during 1 h [25]. Then, the mixture was centrifuged for 5 min at 10,000 rpm in an Eppendorf centrifuge 5804/5804R (Eppendorf SE, Hamburg, Germany). Measurements were also performed on the supernatants separated after digestion, with no solvent addition. Bidistilled water replacing the extract was used as a blank.

Total phenolic content was spectrophotometrically determined following the Folin-Ciocalteau method [29]. An aliquot of 0.125 mL of the previously obtained extract was mixed with 0.5 mL of bidistilled water and 0.125 of the Folin–Ciocalteau reagent (Scharlab S.L., Barcelona, Spain). The mixture was kept in darkness for 6 min, and then 1.25 mL of a 7% sodium carbonate solution and 1 mL of double distilled water were added. After 90 min in darkness, absorbance was measured at 760 nm with a spectrophotometer (Helios Zeta UV/Vis, Thermo Fisher Scientific Inc., Waltham, MA, USA). Results were expressed in mg of gallic acid equivalents (GAE) (purity ≥ 95%, Sigma-Aldrich, St. Louis, MO, USA) per g of dry matter (dm).

Total flavonoid content was determined by following the colorimetric method of aluminium chloride [30]. In total, 1.5 mL of the extract was mixed with 1.5 mL of 2% (*w*/*v*) aluminium chloride (Scharlab S.L., Barcelona, Spain) in a methanol solution. After 10 min of reaction in darkness, the absorbance was measured at 368 nm in a spectrophotometer (Helios Zeta UV/Vis, Thermo Fisher Scientific Inc., Waltham, MA, USA). Flavonoid content was expressed in mg of Quercetin Equivalents (QE) (purity ≥ 95%; Sigma-Aldrich, St. Louis, MO, USA) per g of dry matter (dm).

Antioxidant activity (AO) was measured using the DPPH and ABTS methods, which measure the radical scavenging ability of samples against the free radicals DPPH^+^ (2,2-diphenyl-1-picryl hydrazyl) and ABTS^+^ (2,2-azinobis-3-ethyl benzthiazoline-6-sulphonic acid). Following the method proposed by Brand-Williams et al. [31], 0.1 mL of the extract were made to react with 2 mL of a 0.06 mM solution of DPPH (purity ≥ 98%; Merck KGaA and affiliates, Darmstadt, Germany) in methanol and 0.9 mL of methanol. After 60 min in darkness, absorbance was measured at 517 nm in a spectrophotometer (Helios Zeta UV/Vis, Thermo Fisher Scientific Inc., Waltham, MA, USA). Following the method described by Re et al. [32], ABTS^+^ free radical was first released by mixing a 7 mM solution of ABTS (purity ≥ 98%; VWR International LLC, Radnor, PA, USA) with a 2.45 mM solution of potassium persulfate for 16 h in darkness and at room temperature. Then, the resulting solution was mixed with phosphate buffer (pH 7.4) until reaching an absorbance of 0.70 ± 0.02 at 734 nm. Measurements were carried out by mixing 0.1 mL of the extract with 2.9 mL of the ABTS solution, and the absorbance was read at 734 nm after 7 min of reaction. Antioxidant activities were given in mg of Trolox Equivalent (TE) (purity ≥ 97%; Sigma-Aldrich, St. Louis, MO, USA) per g of dry matter (dm).

#### 2.4.3. Carotenoids Content

The identification and quantification of specific carotenoids were performed using high-performance liquid chromatography (HPLC) following the procedure described by Bunea et al. [33] with some modifications. For this purpose, 1 g of the sample (non-digested or digested powder) was extracted with 25 mL of a 1:1:1 (*v*/*v*/*v*) methanol/ethyl acetate/petroleum ether solution. The mixture was then homogenized for 1 min at 11,000 rpm with a T25D Ultra-Turrax^®^ (IKA^®^-Werke GmbH & Co. KG, Staufen, Germany), vortexed for 1 min (Reax top, Heidolph Instruments GmbH & Co. KG, Schwabach, Germany) and centrifuged (Eppendorf^®^ Centrifuge 5804R, Hamburg, Germany) at 10,000 rpm for 10 min at 10 °C. The supernatant was introduced in a separation funnel. This process was repeated as many times as necessary in order to recover all the carotenoids from the samples, i.e., until a colourless supernatant was obtained. Then, total collected supernatant was washed several times with 100 mL of saturated NaCl saline solution, until both the aqueous and etheric phases were clear. The carotenoid-rich phase (etheric) was dried over anhydrous sodium sulphate and evaporated at 35 °C under vacuum in an Hei-VAP Core rotary evaporator (Heidolph Instruments GmbH & Co., Schwabach, Germany). Finally, the residue was resuspended in 1.5 mL of ethyl acetate.

HPLC analyses were performed with an Alliance 2995 system with DAD detector (Waters, Milford, MA, USA). The chromatographic separation of the compounds was achieved by using a reverse phase Luna^®^ C18 column (250 × 4.6 mm, 5 µm) (Phenomenex, Torrance, CA, USA). Elution was carried out at 25 °C with a mobile phase consisted of mixtures of acetonitrile: water (9:1, *v*/*v*) with 0.25% triethylamine (phase A) and ethyl acetate with 0.25% triethylamine (phase B). The gradient was generated by decreasing phase A from 90 to 50% from 0 to 10 min, to continue decreasing it from 50 to 10% at 20 min, with a flow rate of 1 mL/min. Individual carotenoids selected for their identification and quantification were the most abundant in orange carrot: α-carotene (purity ≥ 95%), β-carotene (purity ≥ 95%), lutein (purity ≥ 96%) and lycopene (purity ≥ 85%), all of them from Merck KGaA and affiliates (Darmstadt, Germany) [3,13,34]. Calibration curves of external standards were prepared in order to identify and quantify the corresponding retention times in the chromatograms, monitored at 450 nm.

### 2.5. Statistical Analysis

The results were statistically analysed using Statgraphics Centurion XVI (Centurion XVII.I version, StatPoint Technologies, Inc., Warrenton, VA, USA) with a confidence level of 95% (*p*-value ≤ 0.05). One-way ANOVA and Multifactor ANOVA were performed to the processing of data. Variables relationships were assessed by means of a Pearson’s product moment correlations test. All the analytical tests described were carried out at least in triplicate.

## 3. Results and Discussion

### 3.1. Physicochemical Properties of Carrot Waste Powder

Water activity, water content and the total soluble solids content of carrot waste powders are summarized in Table 1. Moisture content (87.32 ± 0.02 g_w_/100 g) and water activity (0.996 ± 0.004) of the raw materials were in a range which implied high perishability. Dehydration allowed to reduce carrot water activity below the target value of 0.3, thus ensuring stability [9]. Pre-processing stages may have different impact on drying behaviour and final moisture content depending on the structure of the raw material [25]. Compared to chopping, when drying was conducted at 70 °C, grinding prior to air-drying resulted in a greater moisture content drop for a similar decrease in the water activity. Similar results had been previously obtained by Bas-Bellver et al. [25]. This would indicate that water outflow would have been favoured by the more intense rupture of structures and an increased exchange surface between the solid and the drying air [35]. In contrast, this difference was not observed at 60 °C, when similar final moisture contents were reached for both chopped and ground samples. Case-hardening, which might have occurred at higher temperatures, could have originated an increased internal resistance to water transport [36], this being more significant on larger pieces.

FD powders showed the highest soluble solids content. This is in line with previous results [25], and might be due to the more porous structure resulting from this dehydration process, which, indeed, favours further milling. According to Xiao et al. [37], HAD promotes a more rigid and compact structure, which is especially evident in the outermost layers of the solid. Conversely, ice crystals formation during the freezing process and subsequent sublimation leads to a more porous and fragile structure [38], which increases the surface exposed to extraction and leads to greater fibre breakage [39]. These facts agree with the observed by Yi et al. [40] in mango, pitaya and papaya slices dehydrated by HAD and FD, or Owusu et al. [41] in tomato slices dried at different temperatures. Presumably, a more intense breakage of fibres into simpler units might also contribute to higher soluble solids values. The interdependence between grinding and drying processes and their relationship with the final characteristics of the powders have been previously reported [25,35,42].

Particle size characteristics of powders, as obtained by the wet and dry procedures, are summarized in Table 2. Applying the dry procedure is interesting when incorporating the powder to a solid formulation; in contrast, the wet procedure provides useful information when powders are included to a liquid formulation or solubilized in the gastrointestinal fluids during digestion.

Both the previous disruption intensity and the drying technique applied had a significant impact on the particle size of powders. Grinding prior to drying led to finer powders than chopping. This fact could be due to a more brittle dried product because of a more homogenous water outflow during HAD. When very low moisture contents are reached, more homogeneous drying rates imply the transition of the entire material into the glassy state; in contrast, fast drying rates causing crusting phenomena (case-hardening) implies rubbery cores with an increased moisture content, which reduces milling efficiency [36]. These results are in line with other studies in which it has been evidenced that milling prior to drying determines particle size characteristics [25,35].

**Figure 1:**
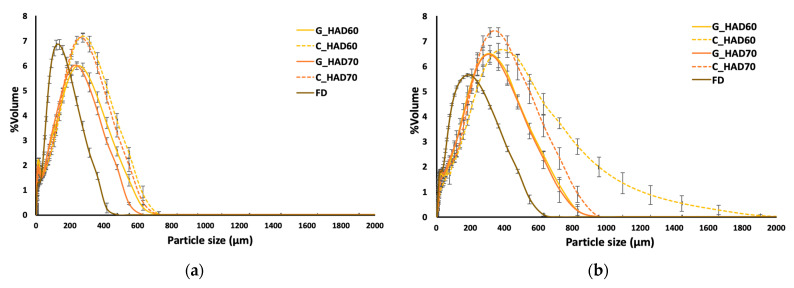
Particle size distribution of carrot residue powders. (**a**) Determination by the dry method. (**b**) Determination by the wet method. Error bars are the standard deviation of five replicates. HAD: hot-air drying at 60 and 70 °C, FD: freeze-drying; C: chopped; G: ground.

Particle size distribution curves are shown in Figure 1. As observed, FD powders exhibited smaller particle sizes, which could be related to the higher brittleness of the porous structure generated in FD products, which facilitates milling as compared to the more compact structure of HAD products [38]. As deduced from the span and the particle size distribution patterns, the wet procedure distributions shift to the right and widen compared with the dry procedure distributions, which indicates the solubilization of finer particles in the water used as the dispersing agent. Additionally, when dispersed in a liquid medium, aggregates may be formed, and thus particle size increase.

### 3.2. Antioxidant Properties of Fresh and Powdered Carrot Waste

Table 3 shows total phenol and flavonoid content, as well as antioxidant capacity (DPPH and ABTS methods) of carrot waste powders and raw carrot waste.

Different processing treatments had a different impact on total phenolics (Table 3). In the available literature, there is evidence that dehydration may have a negative impact on total phenolics, as in Macura et al. [43] who reported a decrease in phenolics in hot-air dried purple carrot samples. Other authors [44], however, have reported an increase in phenolics in hot-air dried and freeze-dried products. In the present study, significant losses of phenolic compounds in FD samples have been observed. This could be attributed to the fact that, once produced, low-moisture FD products are generally more susceptible to oxidation because of their porous structure [45]. In HAD samples, however, losses due to temperature or oxygen exposure during treatment could have been balanced by different biochemical reactions taking place during dehydration and the consequent formation of new antioxidant compounds, which modify the overall antioxidant activity of the product [46]. For instance, Maillard compounds formed during HAD, together with other compounds such as glucose, fructose, tertiary aliphatic amines, tryptophan and other reducing agents [47], are capable of reacting with the Folin–Ciocalteu reagent [48]. A multifactor ANOVA analysis revealed that both air temperature and grinding prior to drying had a significant effect on total phenolic content. Grinding prior to dehydration causes greater damage to vegetal tissue than chopping, releasing more phenolic compounds which would then be more exposed to oxygen, light, or temperature, and thus degraded [47]. On the other hand, a higher drying temperature might increase the phenol content by reducing the activity of certain enzymes capable of degrading phenolic compounds [49], or as a consequence of the generation of new phenolic substances. Moreover, drying at 70 °C shortens processing times and thus the oxidation risk due to airflow exposure. Regarding total flavonoid content, the only factor having a statistically significant effect was the size of the particles to be dried, with chopping leading to a higher flavonoid content, possibly combined with a decreased in the exposed surface to drying conditions.

Antiradical properties of powders showed a different trend depending on the method used. Dehydration of carrot waste led to a significant decrease in its ability to reduce the DPPH radical, but to a significant increase in the antiradical activity measured by the ABTS method (Table 3). These results are in line with those obtained in a previous study [25]. Regardless of the method used, air-drying, especially at 70 °C and after chopping, led to the highest antioxidant activity values, whereas FD led to the lowest ones. This could be, again, evidencing the release or formation of other compounds with antioxidant properties, or products from Maillard reactions during air-drying [47,48]. In addition, the longer exposure time needed to achieve the target a_w_ when dried at 60 °C would imply a higher incidence of oxidative reactions and lower antioxidant capacity as compared with samples dried at 70 °C. Chopping before air-drying had better results than grinding in terms of antioxidant activity values, although not always significant. Similarly, in brassica powders, regarding disruption pre-treatments, chopping generally led to better antioxidant properties than grinding due to the lower cellular damage, so that phenols and flavonoids remain more protected and less susceptible to oxidation during drying [50].

Correlation tests (Pearson’s product moment correlations) were carried out to unveil the relationship between processing conditions and antioxidant properties measured on the powdered products (Table S1). The analysis evidenced that both pretreatment and drying conditions were significantly correlated with the antioxidant properties of powders. The intensity of the disruption is significantly (*p* < 0.01) and negatively correlated with all the antioxidant properties measured, which confirmed a preference for chopping before drying. Regarding drying conditions, a positive significant correlation was evidenced between drying temperature and phenolics and antioxidant activities using the DPPH and ABTS methods. Particle size was also found to be correlated to processing conditions and antioxidant properties.

### 3.3. Carotenoid Content of Carrot Waste Powders

Table 4 shows the results of the individual and total carotenoid content of the carrot waste powders. The most abundant carotenoid in the products obtained was β-carotene followed by α-carotene, which agrees with previous reports [3,13,34]. As compared with FD powders, HAD showed significantly lower carotenoid content, which had been previously evidenced in carrot samples [4,43], but also in other carotenoid-rich wastes such as tomato peels [51] or lulo bagasse [52]. This might be due to the carotenoids’ sensitivity to heat, oxygen, light and degradation by various enzymes such as lipoxygenase, whose activity is reduced during FD, but favoured with the exposure to oxygen and high temperature, conditions which are characteristic of HAD treatments [14]. The susceptibility of carotenoids to the dehydration techniques applied was different for each individual carotenoid. α-carotene and β-carotene were more sensitive to HAD, whereas lycopene was affected to a lesser extent. On the other hand, FD did not prevent the degradation of lutein, as compared with the other carotenoids analysed. This result agrees with the findings of Zhang et al. [53], who concluded that, among the carotenoids present in carrots, lutein would be more resistant to air-drying treatments.

In air-dried powders, an interaction between temperature and disruption conditions was observed. Except for lycopene, total and individual carotenoid content of samples dried at 70 °C was higher in chopped than in ground samples, but the opposite was observed at 60 °C. A possible explanation for this is that, as evidenced for total phenolic content, grinding releases a greater number of compounds of interest, but implies higher degradation due to an enhanced exposure to drying conditions [54]. In addition, chopped samples underwent longer drying treatments compared with ground when dried at 60 °C, whereas processing time was similar for ground and chopped samples dried at 70 °C. Pearson’s product moment correlations (Table S1) displayed a negative relationship between drying temperature and carotenoid content. Particle size was also correlated with carotenoid content, with the smallest particles giving rise to higher levels of carotenoid content.

### 3.4. Simulated In Vitro Digestion of Carrot Waste Powders

#### 3.4.1. Antioxidant Properties along Simulated *In Vitro* Digestion

Response of antioxidants contained in carrot waste powders to the simulated *in vitro* digestion process is summarized in Table 5. Total phenols, total flavonoids and antioxidant capacity (DPPH and ABTS) were measured before digestion (BD) and after the gastric (GP) and intestinal phases (IP). After digestion, antioxidant properties were measured both in the supernatant (S) and the precipitate (P), as described before.

As observed, phenol and flavonoid contents generally decreased after the gastric stage and increased after the intestinal one. As for antioxidant capacities, the ability to react with DPPH sharply increased after the gastric phase and decreased during the intestinal one, but still the DPPH antioxidant activity after digestion was about 1.5–2-fold higher than before digestion. In contrast, ABTS reactivity decreased significantly in the gastric phase, and slightly increased after the intestinal one. Variability in AO assays results during *in vitro* digestion studies has also been observed by other authors, who attributed these differences to both the different chemical principles in which AO methods are based and the pH of the digestion phases [55].

Regarding the response of phenolic constituents during digestion, simulated *in vitro* digestion has been reported to increase the release of phenolics so that, as digestion progresses, compounds become more available. For instance, Gouw et al. [56] and Nayak et al. [57] attributed the increase in the amount of phenolics after *in vitro* digestion to the untangling of these constituents from dietary fibres due to enzymes action and gastric fluids conditions. Similar results had been evidenced for some fruit pomaces in Nayak et al. [58], in which the higher availability was attributed to the continuous liberation of phenolic compounds from macromolecules during the digestion process, together with polyphenol hydrolysis reactions during digestion. Chen et al. [59] also reported that gastric conditions might improve the extractability of phenolic compounds from fruit pomaces. Changes in the pH of the medium and the interaction with the enzymes involved in digestion promote the release by hydrolysis of compounds bound to the food matrix or the formation of new phenolic compounds which implies changes in the antiradical activities measured [27,48]. Accordingly, recovery indexes after the intestinal phase of *in vitro* digestion have been reported to be higher than 100% for most antioxidant properties in dried vegetables [27,55].

However, other authors have evidenced significant losses in phenols and flavonoids after oral and gastric phases, whereas biochemical conditions in the intestinal phase imply an increase. Conditions in the small intestine (pH and pancreatin) would promote the solubilization of certain phenolic compounds that would be previously linked to macromolecules or present in a reduced form [60]. Moreover, the interactions of phenolic compounds with sugars or other dietary compounds released during digestion could play a protective role in their changes during the digestion process, affecting their solubility and potential bioavailability [42]. The results of the present work are in line with these results, since antioxidant constituents present in the supernatant after the intestinal phase increased in all cases, thus suggesting an increased solubilization of phenolics and other compounds during this stage. In the gastric phase, however, antioxidant compounds were more abundant in the precipitate, which suggests that most of them remained bound to the structure. Thus, during the intestinal phase, the enzymatic activity promoted the breakage of structures and the release of part of the liquid phase trapped in the precipitate, it being then collected as supernatant. Antioxidant compounds released to the liquid phase are considered to be more available; however, those present in the precipitate could also be further liberated continuously and slowly and metabolised by the action of colonic microorganisms [61,62]. In addition, the precipitate also retains part of the liquid phase, these compounds being more accessible than the bound ones.

With regard to the differences in DPPH and ABTS antioxidant assays results during *in vitro* digestion, it has been mentioned that differences between both methods could be explained in terms of the different chemical principles in which they are based and the pH of the digestion phases [55]. Nevertheless, differences between DPPH and ABTS assays can also be attributed to the main phenolic constituents in the sample. According to Platzer et al. [63], the AO activity measured depend on multiple criteria and differ between methods: for instance, results in the DPPH assay depend mainly on the number of OH groups and Bors 1 and 3 criteria, whereas there is no clear relationship between the results in the ABTS assay and the number of OH groups and the Bors criteria are much less important. These authors evidenced the variability in the ability to react of the different phenolic constituents so that hydroxycinnamic acids, phenolic acids most abundant in carrot waste [64], achieved higher values than hydroxybenzoic acids in the ABTS assay, whereas this was not the case in the DPPH assay. This could justify the lower values obtained with the DPPH method in the present work. On the other hand, the ABTS assay is very sensitive to pH changes [65], such as the ones occurring during *in vitro* digestion, which could explain the sharp reduction observed in the results after the oral and gastric phases, as well as the different trends observed between the DPPH and ABTS methods. Moreover, regarding antioxidant reactivity, the pH of a substance might modify the compounds’ reactivity and alter their biological reactivity, which could be different in the gastric and duodenal phases.

Focusing on the drying treatments applied, FD powders showed the lowest values at the end of both the gastric and the intestinal phases, for all the antioxidant properties analysed, as they presented the lowest initial values too. However, recovery indexes were very high for freeze-dried powders, suggesting an increased extractability of antioxidant constituents due to simulated *in vitro* digestion. In fact, FD, G_HAD60 and G_HAD70 powders exhibited the highest BI and RI after the intestinal stage. One main reason for this could be the smaller particle size of these powders, which implied a greater contact surface between the sample and the intestinal fluid and an enhanced action of pH and enzymes, thus increasing the extraction and solubilisation of antioxidant compounds [42,66]. Differences in antioxidant properties between both methods used could be explained in terms of the different chemical principles in which they are based and the pH of the digestion phases [55].

#### 3.4.2. Carotenoid Release along Simulated *In Vitro* Digestion

Individual and total carotenoid contents of raw wastes and powders, determined at the end of each stage of the *in vitro* digestion process, as well as the corresponding Recovery Index (RI) values are shown in Table 6. According to the RI values, *in vitro* simulated digestion made both individual and total carotenoids more available.

Throughout the powders’ digestion, an increased release of individual carotenoids was generally observed after IP as compared with GP. This had been also evidenced in persimmon peel powders by Bas-Bellver et al. [42], and in seed-used pumpkin powders by Lyu et al. [67]. In contrast, the opposite was found in raw carrot for β-carotene and α-carotene content, which had also been corroborated by other authors in blended fruit juice [68], or raw carrot juice, but not in raw grated carrot [69]. These results would suggest that matrix transformations during powder manufacturing, pre-treatment and drying, would have favoured the release and solubilisation of carotenoids otherwise linked to the food matrix [18]. In fact, raw samples showed the lowest RI values, which suggests a poorer release of carotenoids during the simulated *in vitro* digestion process or degradation of part of the carotenoids originally present in the raw carrot waste.

Multifactor ANOVA performed on total carotenoid content of HAD powders after *in vitro* digestion revealed that both the drying temperature and the milling intensity had a statistically significant effect (*p* < 0.05). Increasing the drying temperature had a positive impact on total carotenoid release, which could be due to its effects on the dried carrot structure and consequent impact of powder particle size characteristics. Other authors [21] have evidenced a positive effect of cooking on the release of carrot carotenes during digestion and attributed this improvement to structural modifications which occurred during cooking. On the other hand, ground samples exhibited higher total carotenoid release than chopped ones, which became more evident when drying at 60 °C than at 70 °C. Again, the degree of cellular rupture could be involved in an increased carotenoids release. In fact, other authors have reported that mechanical disruption of vegetal matrix could be the main factor for carotenoid release and accessibility to digestion fluids [18,70,71]. The cell wall acts as the main structural physical barrier that drives the release of carotenoids [53], for which disruption of the food matrix is the first step in the absorption process [21,70]. Cell wall integrity is related to particle size, and it is also influenced by interactions between the structural compounds that conform the food matrix [71]. Therefore, disruption favours the release of a greater number of bioactive compounds, allowing their extraction from the food matrix [54]. Among air-dried samples, recovery indexes were higher for those powders obtained at (70 °C). Recovery indexes reveal that a more intense disruption pre-treatment, together with dehydration, and especially at the highest temperature assayed, improves carotenoid extraction during *in vitro* digestion.

In contrast, FD powders presented the lowest RI values for total carotenoids at the end of the intestinal stage, in spite of them having the highest total carotenoid content in the final digestion process. FD powders initially presented higher carotenoid content than HAD ones, most likely due to treatment conditions and the resulting structure and particle size, which would have facilitated carotenoids extraction. The lower temperature and less exposure to oxygen during FD could have also contributed to the preservation of the carotenoids. On the contrary, HAD powders presented lower initial carotenoid content, either because they had been degraded or because they remained trapped in the structure, so that the action of digestion fluids and their enzymes was more relevant for their release and bioaccessibility. Additionally, the presence of antioxidant compounds, which was higher in HAD powders than in FD, might have prevented carotenoid degradation during digestion, as suggested in the literature [18].

#### 3.4.3. Interaction with Lipids during *In Vitro* Digestion

Processing and interaction with other compounds present in the food are considered determining factors for carotenoids release and bioaccessibility. Mechanical disruption, the application of heat treatments and the addition of fat during the processing of fruit and vegetables have been reported to play a significant role in the release of carotenoids [72,73]. Due to its lipophilic character, the amount of fat, as well as the type of fat consumed, influences the bioavailability of carotenoids from natural sources [73,74,75]. The effect of dispersing the powders in water, an oil-in-water emulsion (10% oil), or sunflower oil (1:1 *v*/*v* ratio), on carotenoid release during the *in vitro* digestion process was evaluated.

**Figure 2:**
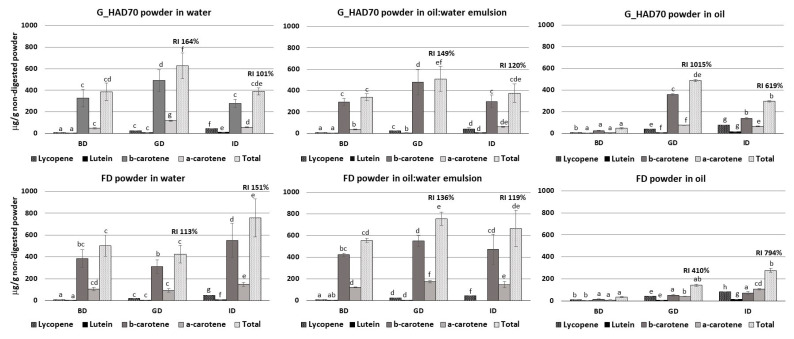
Total and individual carotenoid content (μg/g of non-digested powder) of selected powders mixed with water, 10% oil-in-water emulsion, or oil. Values of undigested samples (before digestion (BD)) and digested ones (after the gastric (GP) and after the intestinal phase (IP)) are given. Recovery index (RI) for total carotenoids after the gastric and intestinal phases are given in percentage. G_HAD70: ground and air-dried at 70 °C, FD: ground and freeze-dried. Values plotted correspond to mean ± standard deviation of four measurements. ^a,b,c,d,e,f,g,h.^ Different superscript letters for a similar carotenoid and treatment (G_HAD70 or FD) indicate statistically significant differences at the 95% confidence level (*p*-value < 0.05).

The different media in which powders were dispersed had a statistically significant impact on carotenoids measurements (Figure 2). It was evidenced that, as compared to powders alone, the addition of water and the oil-in-water emulsion implied an increased carotenoid content identified in non-digested samples. This fact could suggest that incorporating the powders into a liquid matrix would favour the solubilization and extraction of carotenoids otherwise bound to the powder structure. In contrast, when powders were mixed with oil with no water addition, the number of determined carotenoids decreased significantly, especially α- and β-carotene, and more remarkably in the FD powder. Similar results have been reported by other authors, who have found lower carotenoid content when increasing the amount of oil, and have attributed this result to limitations in the method used [21]. In our case, poor recovery of carotenoids when powders were dispersed in the oil matrix was also evidenced.

Focusing on the digestion process, the total carotenoid content in the digest of powders dispersed in water or oil-in-water emulsion were in the range of the determined powders that were directly digested, not dispersed in any media other than digestion fluids. When dispersed in the emulsion, there was an increase in some individual carotenoids during digestion, especially in FD powders, a fact which has also been observed by other authors [48,67,71,75]. Other carotenoids, however, decreased when powders were dispersed in the emulsion, which was even more remarkable in the case of powders dispersed in oil. As explained in the previous paragraph, limitations in the methodology used could be partially responsible for this result; nevertheless, our results also evidence poor solubilization of carotenoids in the sunflower oil used as the dispersing medium. However, the recovery indexes obtained after digestion of the powders dispersed in oil suggest that the *in vitro* digestion process favours the micellarisation of carotenoids, as compared with the non-digested samples. In fact, *in vitro* digestion significantly increased the number of carotenoids quantified, compared with the initial (non-digested) samples when adding oil, which suggests that the digestion fluids together with the stirring provided during the digestion procedure favours carotenoids micellarisation and quantification, and thus, carotenoids’ bioaccessibility [53,67].

Recovery indexes for total carotenoid content showed an increased release of carotenoids during digestion, for all three dispersing media analysed. Drying technique, either air-drying or freeze-drying, did have an impact on carotenoid release during digestion. As deduced from the RI, in G_HAD70 powders, the level of carotenoids released increased after the GP and decreased after the IP, for the three matrices tested. In contrast, carotenoids in FD powders were released throughout the process and increased after both GP and IP phases, exhibiting, in this case, a higher amount at the end of the process, especially in powders mixed with water or emulsion. This progressive release of carotenoids in FD samples during the *in vitro* digestion process has also been observed in powders digested with no medium addition (Table 6), and has been related to particle size and powder structure. Other authors have evidenced that a smaller particle size improves carotenoid release, such as in the study of Lyu et al. [67] on pumpkin seed powders; Zhang et al. [53] on dried carrot, sweet potato, yellow bell pepper and broccoli; or Moelants et al. [71] on carrot- and tomato-derived particles.

## 4. Conclusions

The valorisation of carrot residues and discards as functional powdered ingredients is an interesting alternative towards the development of more sustainable food systems and the concept of sustainable healthy diets. As evidenced, disruption and drying can be combined to obtain powders with appropriate a_w_ characteristics and interesting nutritional properties. Both dehydration and disruption treatments influenced physicochemical and antioxidant properties of the powders obtained, as well as carotenoid content. In general, air-drying produced powders with improved antioxidant properties, whereas freeze-drying yielded finer powders with a higher carotenoid content.

Simulated *in vitro* digestion studies revealed that digestion helps to release bioactive compounds which are bound to the powder structure. The response to digestion is greatly influenced by processing conditions, which implied different physical characteristics of powders; finer ones such as freeze-dried powders were found to release more bioactive constituents during digestion. In addition to processing conditions, fat co-ingestion also influenced the powders’ response to digestion, recovery indexes being increased in the case of powders dispersed in oil. As simulation digestion proceeds, digestion fluids together with stirring promote the breakage of structures as well as carotenoid release and micellarisation.

Future research should explore ensuring emulsification to better assess the impact of fat co-ingestion on the bioaccessibility of carotenoids. Hence, other emulsions (different fats, different fat concentrations, other emulsification procedures) should be tested. In addition, it would be convenient to modify the digestion protocol by increasing the amount of bile salts, thus simulating the increased level of bile salt secretion during the co-ingestion of fats. This is expected to increase the number and size of micelles, facilitating the solubilisation of carotenoids.

## Figures and Tables

**Table 1:** Water activity (a_w_), moisture content (x_w_) and soluble solids content (X_ss_) of carrot residue powders. G: ground, C: chopped; HAD: hot-air drying at 60 and 70 °C, FD: freeze drying. Mean ± standard deviation of three independent measurements.

Sample	a_w_	x_w_ (g_w_/100 g)	X_ss_ (g_ss_/g_dm_)
G_HAD60	0.254 ± 0.008 ^b^	2.9 ± 0.4 ^b^	0.667 ± 0.017 ^a^
C_HAD60	0.239 ± 0.010 ^ab^	2.96 ± 0.10 ^b^	0.659 ± 0.017 ^a^
G_HAD70	0.236 ± 0.011 ^a^	1.6 ± 0.3 ^a^	0.685 ± 0.011 ^ab^
C_HAD70	0.240 ± 0.005 ^ab^	3.26 ± 0.12 ^b^	0.685 ± 0.012 ^bc^
FD	0.236 ± 0.007 ^a^	2.80 ± 0.11 ^b^	0.724 ± 0.018 ^c^

^a,b,c^ Different superscript letters in the same column indicate statistically significant differences at the 95% confidence level (*p*-value < 0.05).

**Table 2:** Particle size characteristic parameters obtained by the wet and dry procedures: equivalent volume diameter D [4,3], surface area mean diameter D [3,2], percentiles d_10_, d_50_, and d_90_. HAD: hot-air drying at 60 and 70 °C, FD: freeze-drying; C: chopped; G: ground. Mean ± standard deviation of five replicates.

	**DRY PROCEDURE**				
	**D[4,3]**	**D[3,2]**	**d_10_**	**d_50_**	**d_90_**
G_HAD60	171 ± 6 ^c^	34.6 ± 1.3 ^b^	11.6 ± 0.3 ^b^	137 ± 7 ^c^	391 ± 10 ^c^
C_HAD60	210 ± 6 ^e^	51.2 ± 1.5 ^c^	17.8 ± 0.5 ^d^	190 ± 7 ^e^	442 ± 12 ^e^
G_HAD70	155 ± 3 ^b^	26.0 ± 0.6 ^a^	8.6 ± 0.2 ^a^	126 ± 5 ^b^	358 ± 5 ^b^
C_HAD70	200 ± 6 ^d^	50 ± 3 ^c^	17.36 ± 0.7 ^d^	180 ± 7 ^d^	423 ± 9 ^d^
FD	124 ± 3 ^a^	33.1 ± 1.7 ^b^	12.5 ± 0.8 ^c^	107 ± 3 ^a^	258 ± 6 ^a^
	**WET PROCEDURE**				
	**D[4,3]**	**D[3,2]**	**d_10_**	**d_50_**	**d_90_**
G_HAD60	245 ± 24 ^b^	53 ± 3 ^c^	20.6 ± 1.2 ^b^	209 ± 20 ^b^	530 ± 54 ^b^
C_HAD60	348 ± 37 ^d^	78.8 ± 1.9 ^e^	35.4 ± 1.1 ^d^	292 ± 16 ^d^	723 ± 97 ^c^
G_HAD70	228 ± 8 ^b^	48 ± 2 ^b^	18.8 ± 1.1 ^a^	194 ± 9 ^b^	500 ± 23 ^b^
C_HAD70	273 ± 18 ^c^	67.1 ± 1.9 ^d^	28.8 ± 0.8 ^c^	249 ± 16 ^c^	562 ± 37 ^b^
FD	156 ± 2 ^a^	39.0 ± 0.4 ^a^	15.8 ± 0.3 ^a^	123.6 ± 1.4 ^a^	350 ± 6 ^a^

^a,b,c,d,e^ Different superscript letters in the same column indicate statistically significant differences at the 95% confidence level (*p*-value < 0.05).

**Table 3:** Total phenol content, total flavonoid content and antioxidant capacity by the DPPH and ABTS methods. HAD: hot-air drying at 60 and 70 °C, FD: freeze-drying; C: chopped, G: ground. Mean ± standard deviation of the three measurements.

Sample	Total Phenols (mg GAE/g_dm_)	Total Flavonoids (mg QE/g_dm_)	DPPH (mg TE/g_dm_)	ABTS (mg TE/g_dm_)
Carrot waste	2.03 ± 0.09 ^c^	1.210 ± 0.012 ^a^	4.28 ± 0.04 ^e^	14.0 ± 0.6 ^a^
G_HAD60	1.53 ± 0.12 ^b^	1.24 ± 0.06 ^ab^	1.90 ± 0.12 ^b^	55 ± 2 ^b^
C_HAD60	2.06 ± 0.16 ^c^	1.464 ± 0.003 ^c^	2.1 ± 0.2 ^c^	57.5 ± 1.4 ^b^
G_HAD70	2.004 ± 0.013 ^c^	1.27 ± 0.03 ^b^	1.69 ± 0.10 ^b^	62 ± 3 ^c^
C_HAD70	2.42 ± 0.15 ^d^	1.45 ± 0.02 ^c^	2.65 ± 0.11 ^d^	64.8 ± 1.7 ^c^
FD	0.74 ± 0.14 ^a^	1.26 ± 0.03 ^ab^	1.01 ± 0.11 ^a^	16.9 ± 0.3 ^a^

^a,b,c,d,e.^ Different superscript letters in the same column indicate statistically significant differences at the 95% confidence level (*p*-value < 0.05).

**Table 4:** Carotenoid content of carrot waste powders. HAD: hot-air drying at 60 and 70 °C, FD: freeze-drying; C: chopped, G: ground. Mean ± standard deviation.

Sample	Lycopene (µg/g_dm_)	Lutein (µg/g_dm_)	β-Carotene (µg/g_dm_)	α-Carotene (µg/g_dm_)	Total (µg/g_dm_)
G_HAD60	3.19 ± 0.02 ^c^	1.29 ± 0.04 ^c^	59 ± 15 ^c^	9.99 ± 0.10 ^b^	73 ± 15 ^c^
C_HAD60	2.64 ± 0.07 ^b^	0.92 ± 0.15 ^a^	29.1 ± 0.2 ^a^	4.08 ± 0.04 ^a^	37.0 ± 0.6 ^a^
G_HAD70	3.08 ± 0.03 ^c^	1.036 ± 0.011 ^ab^	42.2 ± 1.7 ^b^	9.4 ± 1.2 ^b^	56 ± 3 ^b^
C_HAD70	2.517 ± 0.011 ^a^	1.142 ± 0.006 ^bc^	52.5 ± 0.8 ^bc^	12.04 ± 0.05 ^b^	67.8 ± 0.6 ^c^
FD	4.77 ± 0.09 ^d^	1.147 ± 0.005 ^bc^	221 ± 4 ^d^	59 ± 2 ^c^	286 ± 2 ^d^

^a,b,c,d^ Different superscript letters in the same column indicate statistically significant differences at the 95% confidence level (*p*-value < 0.05).

**Table 5:** Total phenol content, total flavonoid content and antioxidant capacity (DPPH and ABTS) of carrot waste powders before digestion (BD), after the gastric phase (GP) in the supernatant (S) and precipitate (P), and after the intestinal phase (IP) in the supernatant (S) and precipitate (P) of the *in vitro* digestion process. Results are given per gram of non-digested sample. Recovery (RI) and bioaccesibility (BI) indexes are expressed in percentage. Mean ± standard deviation of three measurements.

	Sample	BD	Gastric Phase (GP)			Intestinal Phase (IP)			
			S	P	TOTAL (%RI)	S	P	TOTAL (%RI)	%BI
Total phenol content (mg GAE/g)	G_HAD60	1.48 ± 0.11 ^b^	0.24 ± 0.02 ^b^	0.68 ± 0.10 ^b^	0.93 ± 0.10 ^b^ (63% ± 7 ^c^)	1.01 ± 0.04 ^b^	0.35 ± 0.02 ^a^	1.37 ± 0.05 ^b^ (92% ± 3 ^b^)	100 ± 4 ^c^
	C_HAD60	2.00 ± 0.15 ^c^	0.259 ± 0.011 ^b^	0.7 ± 0.2 ^b^	0.96 ± 0.18 ^b^ (48% ± 9 ^b^)	1.22 ± 0.04 ^c^	0.42 ± 0.05 ^a^	1.64 ± 0.04 ^c^ (82% ± 2 ^ab^)	82 ± 3 ^b^
	G_HAD70	1.972 ± 0.013 ^c^	0.49 ± 0.02 ^d^	1.41 ± 0.08 ^c^	1.89 ± 0.10 ^c^ (96% ± 5 ^d^)	2.05 ± 0.10 ^d^	0.85 ± 0.12 ^b^	2.9 ± 0.2 ^d^ (147% ± 10 ^c^)	140 ± 7 ^d^
	C_HAD70	2.34 ± 0.14 ^d^	0.46 ± 0.02 ^c^	1.8 ± 0.2 ^d^	2.2 ± 0.2 ^d^ (95% ± 8 ^d^)	0.84 ± 0.05 ^a^	0.96 ± 0.15 ^b^	1.81 ± 0.10 ^c^ (77% ± 4 ^a^)	66 ± 4 ^a^
	FD	0.72 ± 0.13 ^a^	0.157 ± 0.005 ^a^	0.052 ± 0.014 ^a^	0.21 ± 0.02 ^a^ (29% ± 3 ^a^)	0.81 ± 0.03 ^a^	0.33 ± 0.03 ^a^	1.14 ± 0.6 ^a^ (158% ± 8 ^c^)	158 ± 5 ^e^
Total flavonoid content (mg QE/g)	G_HAD60	1.20 ± 0.06 ^a^	0.44 ± 0.05 ^b^	0.245 ± 0.008 ^b^	0.69 ± 0.05 ^b^ (57% ± 4 ^c^)	0.210 ± 0.005 ^a^	1.22 ± 0.08 ^d^	1.43 ± 0.09 ^d^ (118% ± 7 ^e^)	25.5 ± 0.6 ^a^
	C_HAD60	1.421 ± 0.003 ^c^	0.24 ± 0.08 ^a^	0.272 ± 0.009 ^b^	0.51 ± 0.09 ^a^ (36% ± 6 ^a^)	0.279 ± 0.013 ^ab^	0.47 ± 0.04 ^b^	0.75 ± 0.04 ^b^ (53% ± 2 ^b^)	26.4 ± 1.2 ^a^
	G_HAD70	1.25 ± 0.03 ^b^	0.39 ± 0.04 ^b^	0.73 ± 0.05 ^c^	1.13 ± 0.06 ^c^ (90% ± 5 ^d^)	0.83 ± 0.03 ^d^	0.462 ± 0.009 ^b^	1.30 ± 0.02 ^c^ (104% ± 2 ^d^)	90 ± 3 ^c^
	C_HAD70	1.40 ± 0.02 ^c^	0.3813 ± 0.0011 ^b^	1.137 ± 0.011 ^d^	1.518 ± 0.010 ^d^ (108.6% ± 0.7 ^e^)	0.59 ± 0.04 ^c^	0.658 ± 0.012 ^c^	1.25 ± 0.04 ^c^ (89% ± 3 ^c^)	77 ± 5 ^b^
	FD	1.23 ± 0.03 ^ab^	0.44 ± 0.05 ^b^	0.090 ± 0.013 ^a^	0.53 ± 0.04 ^a^ (44% ± 3 ^b^)	0.28 ± 0.074 ^b^	0.27 ± 0.05 ^a^	0.55 ± 0.05 ^a^ (45% ± 4 ^a^)	33 ± 8 ^a^
DPPH (mg TE/g)	G_HAD60	1.85 ± 0.12 ^b^	0.25 ± 0.03 ^c^	7.7 ± 0.9 ^b^	7.9 ± 0.9 ^b^ (428% ± 49 ^a^)	0.64 ± 0.07 ^a^	3.1 ± 0.4 ^b^	3.3 ± 0.7 ^b^ (181% ± 38 ^ab^)	51 ± 6 ^a^
	C_HAD60	2.1 ± 0.2 ^c^	0.180 ± 0.003 ^b^	8.5 ± 0.6 ^bc^	8.6 ± 0.6 ^bc^ (417% ± 28 ^a^)	0.79 ± 0.05 ^b^	2.9 ± 0.4 ^b^	3.7 ± 0.3 ^bc^ (179% ± 16 ^a^)	51 ± 3 ^a^
	G_HAD70	1.67 ± 0.10 ^b^	0.319 ± 0.003 ^d^	8.3 ± 0.3 ^bc^	8.6 ± 0.4 ^bc^ (519% ± 21 ^b^)	1.37 ± 0.05 ^c^	2.90 ± 0.13 ^b^	4.27 ± 0.08 ^c^ (256% ± 5 ^c^)	111 ± 4 ^c^
	C_HAD70	2.56 ± 0.10 ^d^	0.318 ± 0.004 ^d^	8.8 ± 0.5 ^c^	9.1 ± 0.5 ^c^ (356% ± 19 ^a^)	0.63 ± 0.06 ^a^	3.3 ± 0.8 ^b^	3.9 ± 0.8 ^bc^ (153% ± 31 ^a^)	45 ± 4 ^a^
	FD	0.99 ± 0.11 ^a^	0.100 ± 0.004 ^a^	6.1 ± 0.6 ^a^	6.3 ± 0.6 ^a^ (634% ± 64 ^c^)	0.53 ± 0.10 ^a^	1.61 ± 0.11 ^a^	2.1 ± 0.2 ^a^ (217% ± 20 ^b^)	76 ± 14 ^b^
ABTS (mg TE/g)	G_HAD60	53 ± 2 ^b^	2.25 ± 0.03 ^d^	2.6 ± 0.9 ^a^	4.9 ± 0.9 ^a^ (9.2% ± 1.7 ^a^)	8.88 ± 0.09 ^c^	4.0 ± 0.5 ^a^	12.9 ± 0.4 ^b^ (24.4% ± 0.8 ^b^)	24.4 ± 0.2 ^b^
	C_HAD60	55.8 ± 1.3 ^b^	1.45 ± 0.02 ^b^	4.6 ± 1.3 ^b^	6.0 ± 1.3 ^a^ (11% ± 2 ^a^)	11.0 ± 0.2 ^d^	6.8 ± 0.6 ^bc^	17.8 ± 0.8 ^cd^ (31.9% ± 1.4 ^c^)	26.5 ± 0.5 ^c^
	G_HAD70	61 ± 3 ^c^	2.06 ± 0.03 ^c^	3.6 ± 0.9 ^ab^	5.7 ± 0.9 ^a^ (9.3% ± 1.4 ^a^)	12.3 ± 0.3 ^e^	6.3 ± 1.4 ^b^	18.54 ± 1.13 ^d^ (30.3% ± 1.8 ^c^)	27.0 ± 0.6 ^c^
	C_HAD70	62.6 ± 1.6 ^c^	2.01 ± 0.02 ^c^	3.4 ± 0.4 ^ab^	5.4 ± 0.4 ^a^ (8.6% ± 0.6 ^a^)	7.1 ± 0.3 ^a^	3.7 ± 1.2 ^a^	10.8 ± 1.6 ^a^ (17% ± 2 ^a^)	20.8 ± 0.9 ^a^
	FD	16.4 ± 0.3 ^a^	1.06 ± 0.05 ^a^	15.05 ± 1.12 ^c^	16.11 ± 1.09 ^b^ (98% ± 7 ^b^)	7.5 ± 0.2 ^b^	8.4 ± 0.9 ^c^	15.9 ± 0.9 ^c^ (97% ± 6 ^d^)	64.1 ± 1.4 ^d^

^a,b,c,d,e^ Different superscript letters in the same column for the same property analysed indicate statistically significant differences at the 95% confidence level (*p*-value < 0.05).

**Table 6:** Total and individual carotenoid content (μg/g of non-digested sample) of carrot waste and carrot powders after the gastric phase (GP) and the intestinal phase (IP). Recovery index in percentage (%RI) after GP and IP. HAD: hot-air drying at 60 and 70 °C, FD: freeze-drying; C: chopped, G: ground. Mean ± standard deviation of four replicates.

			Carrot	G_HAD60	C_HAD60	G_HAD70	C_HAD70	FD
Lycopene	GP	µg/g	12.96 ± 0.05 ^e^	11.2 ± 0.2 ^b^	10.54 ± 0.06 ^a^	12.53 ± 0.08 ^d^	13.50 ± 0.04 ^f^	11.76 ± 0.07 ^c^
		%RI	241.2 ± 0.9 ^a^	362 ± 7 ^c^	411 ± 2 ^d^	414 ± 3 ^d^	554.3 ± 1.7 ^e^	253.3 ± 1.5 ^b^
	IP	µg/g	22.22 ± 0.07 ^c^	21.02 ± 0.09 ^a^	21.43 ± 0.05 ^b^	23.13 ± 0.17 ^e^	22.6 ± 0.3 ^d^	22.8 ± 0.5 ^de^
		%RI	413.7 ± 1.3 ^a^	679 ± 3 ^c^	836.7 ± 1.9 ^e^	764 ± 6 ^d^	929 ± 12 ^f^	490 ± 10 ^b^
Lutein	GP	µg/g	4.93 ± 0.03 ^e^	3.2 ± 0.2 ^c^	2.66 ± 0.02 ^b^	3.09 ± 0.06 ^c^	3.68 ± 0.08 ^d^	2.48 ± 0.02 ^a^
		%RI	40.5 ± 0.2 ^a^	258 ± 19 ^c^	297 ± 2 ^d^	303 ± 6 ^d^	333 ± 7 ^e^	222 ± 2 ^b^
	IP	µg/g	5.91 ± 0.10 ^c^	4.83 ± 0.07 ^b^	4.84 ± 0.10 ^b^	4.78 ± 0.14 ^b^	4.72 ± 0.11 ^ab^	4.61 ± 0.09 ^a^
		%RI	48.5 ± 0.9 ^a^	385 ± 6 ^b^	540 ± 12 ^e^	469 ± 14 ^d^	428 ± 10 ^c^	414 ± 8 ^c^
β-carotene	GP	µg/g	611 ± 15 ^f^	182 ± 10 ^b^	138 ± 3 ^a^	415 ± 12 ^d^	443 ± 14 ^e^	331 ± 10 ^c^
		%RI	109 ± 3 ^a^	318 ± 17 ^c^	490 ± 12 ^d^	1000 ± 30 ^f^	878 ± 28 ^e^	154 ± 5 ^b^
	IP	µg/g	350 ± 40 ^b^	436 ± 14 ^c^	100 ± 4 ^a^	422 ± 5 ^c^	456 ± 10 ^c^	528 ± 55 ^d^
		%RI	62 ± 7 ^a^	764 ± 25 ^d^	355 ± 13 ^c^	1017 ± 11 ^f^	905 ±21 ^e^	246 ± 26 ^b^
α-carotene	GP	µg/g	157 ± 9 ^e^	37 ± 3 ^c^	17.5 ± 0.3 ^a^	27.2 ± 0.5 ^b^	14.2 ± 0.5 ^a^	92 ± 5 ^d^
		%RI	98 ± 6 ^a^	383 ± 27 ^e^	441 ± 7 ^f^	294 ± 6 ^d^	122 ± 4 ^b^	160 ± 9 ^c^
	IP	µg/g	128 ± 15 ^d^	52.2 ± 0.9 ^c^	30 ± 2 ^b^	37.6 ± 0.7 ^b^	16.2 ± 0.8 ^a^	166 ± 8 ^e^
		%RI	80 ± 9 ^a^	537 ± 10 ^e^	752 ± 62 ^f^	406 ± 7 ^d^	139 ± 7 ^b^	291 ± 13 ^c^
Total	GP	µg/g	787 ± 27 ^e^	223 ± 12 ^b^	169 ± 3 ^a^	458 ± 13 ^d^	474 ± 14 ^d^	437 ± 11 ^c^
		%RI	106 ± 3 ^a^	328 ± 17 ^c^	470 ± 10 ^d^	835 ± 23 ^f^	723 ± 22 ^e^	157 ± 4 ^b^
	IP	µg/g	505 ± 27 ^b^	514 ± 15 ^b^	156.1 ± 1.6 ^a^	488 ± 4 ^b^	500 ± 11 ^b^	722 ± 61 ^c^
		%RI	68 ± 4 ^a^	723 ± 22 ^d^	435 ± 4 ^c^	890 ± 8 ^f^	762 ± 17 ^e^	260 ± 22 ^b^

^a,b,c,d,e,f.^ Different superscript letters in the same column for the same carotenoid indicate statistically significant differences at the 95% confidence level (*p*-value < 0.05).

## Data Availability

Data are available from the authors upon reasonable request.

## Supplementary Materials

The following supporting information can be downloaded at: https://www.mdpi.com/article/10.3390/foods12040731/s1, Table S1. Pearson’s product moment correlations matrix. Processing variables: disruption intensity, drying temperature; measured variables: particle size, total phenols, total flavonoids, DPPH antioxidant capacity, ABTS antioxidant capacity and total carotenoid content.
