# Supplementary material for: Effect of Processing and In Vitro Digestion on Bioactive Constituents of Powdered IV Range Carrot (Daucus carota, L.) Wastes
Source: Foods. 2023 Feb 7;12(4):731. doi: 10.3390/foods12040731 (PMC9955751; doi:10.3390/foods12040731)
Supplement: Supplementary file 1 [file foods-12-00731-s001.zip › foods-2139367-supplementary.pdf]

Table S1. Pearson's product moment correlations matrix. Processing variables: disruption intensity, drying temperature; Measured variables: particle size, total phenols, total flavonoids, DPPH antioxidant capacity, ABTS antioxidant capacity and total carotenoid content.

	Disruption intensity	*Drying temperature*	*Particle size*	*Phenols*	*Flavonoids*	*DPPH*	*ABTS*	*Carotenoids*
**Disruption intensity**	1							
**Drying temperature**	-0.3932	1						
**Particle size**	-0.8626**	0.6942**	1					
**Phenols**	-0.6792**	0.9122**	0.8117**	1				
**Flavonoids**	-0.9572**	0.3509	0.8183**	0.6156*	1			
**DPPH**	-0.7632**	0.7921**	0.8782**	0.8972*	0.6841**	1		
**ABTS**	-0.4598**	0.9905**	0.7553**	0.9257**	0.4002**	0.8245**	1	
**Carotenoids**	0.4575	-0.9441**	-0.7911**	-0.8670**	-0.4249	-0.7708**	-0.9636**	1

*Indicates significant correlation at the 95% confidence level (p-value<0.05). **Indicates significant correlation at the 99% confidence level (p-value<0.01)
